# Etomidate Is Associated with Higher 30-Day Mortality than Ketamine for Emergency Intubation of COVID-19 Patients: A Propensity-Matched Cohort Study

**DOI:** 10.3390/jcm15135050

**Published:** 2026-06-29

**Authors:** Li-Wei Lin, Tzu-Fu Huang, Chi-Chieh Huang, Chee-Fah Chong

**Affiliations:** 1 Department of Emergency Medicine, Shin Kong Wu Ho-Su Memorial Hospital, No. 95, Wenchang Rd., Shihlin Dist., Taipei 11101, Taiwan; dribliner@gmail.com (L.-W.L.); a005289@ms.skh.org.tw (C.-C.H.); 2 School of Medicine, College of Medicine, Fu Jen Catholic University, No. 510, Zhongzheng Rd., Xinzhuang Dist., New Taipei City 24205, Taiwan; 3 CrazyatLAB (Critical Airway Training Laboratory), Taipei, Taiwan; 4 Department of Research, Shin Kong Wu Ho-Su Memorial Hospital, No. 95, Wenchang Rd., Shihlin Dist., Taipei 11101, Taiwan; gh52025@gmail.com

**Keywords:** COVID-19, Critical Care, etomidate, ketamine, mortality, rapid sequence intubation, adrenal insufficiency

## Abstract

**Background:** COVID-19 causes adrenal injury that may amplify etomidate-induced adrenal suppression. We compared 30-day mortality between etomidate and ketamine for emergency intubation. **Methods:** We conducted a TriNetX-based propensity-matched cohort study of adult COVID-19 patients undergoing emergency intubation with etomidate or ketamine (January 2020–June 2025). The primary outcome was 30-day mortality. Three independent propensity-matched cohorts were created using different vasopressor inclusion criteria: primary (no vasopressor on day −1), hemodynamically stable (no vasopressor on day −1 or day 0), and critically ill (vasopressor on both day −1 and day 0). Sensitivity and negative control analyses addressed confounding. **Results:** Among 9462 primary-cohort pairs, etomidate was associated with higher 30-day mortality (34.43% vs. 31.57%; HR 1.11, 95% CI 1.06–1.17, *p* < 0.001; NNH 35). The association persisted in stable patients (HR 1.16, *p* = 0.013) and in critically ill patients (HR 1.15, *p* < 0.001). Mortality hazard ratios rose progressively with longer follow-up (day 0–7: HR 1.05; day 0–30: HR 1.11), indicating a delayed effect. In stable patients, etomidate was associated with higher vasopressor initiation (HR 1.15, *p* = 0.005); the between-group difference appeared early (4.41% by day 1) and narrowed thereafter, while the mortality difference emerged later and widened through day 30. **Conclusions:** Etomidate was consistently associated with higher 30-day mortality in COVID-19 patients (HR 1.11–1.16), unlike the results seen in general critical illness. The temporal pattern of early vasopressor divergence and late mortality divergence, together with consistency across hemodynamic strata, supports disease-specific adrenal vulnerability rather than acute peri-intubation harm. Ketamine may be the preferred induction agent in COVID-19 patients without contraindications.

## 1. Introduction

COVID-19 (Coronavirus Disease 2019) is associated with hypothalamic–pituitary–adrenalhypothalamic-pituitary-adrenal (HPA) axis dysregulation [1,2]. SARS-CoV-2 (Severe Acute Respiratory Syndrome Coronavirus 2) exhibits direct adrenal tropism, with autopsy studies demonstrating adrenal viral replication accompanied by inflammation, widespread microthrombosis, and severe adrenal injury [3]. Histopathological examination reveals inflammatory cell infiltration with CD3+ and CD8+ T-lymphocytes [4], and microscopic adrenal lesions including ischemic necrosis, cortical lipid degeneration, and hemorrhage were identified in 43% of fatal COVID-19 cases in one autopsy series [5]. On imaging studies, acute adrenal infarction was detected in 23% of severe COVID-19 patients on chest CT evaluation and associated with higher ICU admission rates and prolonged ICU stays [6].

The clinical significance of this adrenal involvement is substantial. During acute infection, patients mount a dysregulated cortisol stress response characterized by a bidirectional pattern: elevated baseline cortisol concentrations (≥27 μg/dL) independently predict mortality [7], yet paradoxically, hypocortisolism (<11 μg/dL) is observed in up to 64% of hospitalized patients 1–2 days after admission [8]. Low cortisol levels are associated with significantly higher mortality risk; each unit increase in cortisol is associated with a 26% reduction in mortality risk [9]. This complex dysregulation—where both excessive and insufficient cortisol predict poor outcomes—reflects unique HPA-axis perturbations characteristic of COVID-19.

Importantly, adrenal dysfunction in COVID-19 may be subclinical and undetected during acute illness. Longitudinal studies demonstrate that 42% of COVID-19 patients with confirmed adrenal insufficiency at follow-up had not met standard diagnostic criteria during their acute hospitalization [10], suggesting that subclinical HPA-axis compromise may already be present at the time of emergency intubation. The clinical importance of preserved adrenal function during acute COVID-19 is underscored by a large Swedish population-based study demonstrating that patients with pre-existing adrenal insufficiency had markedly increased COVID-19-related mortality (hazard ratio 2.29, 95% CI 1.60–3.28) compared to matched controls [11], raising concerns that any additional adrenal suppression could be particularly detrimental in this population.

The selection of induction agents for rapid sequence intubation in critically ill patients has evolved considerably in recent years. Both etomidate and ketamine are favored for their hemodynamic stability [12,13,14]. Etomidate, a short-acting non-barbiturate hypnotic, provides exceptional cardiovascular stability but inhibits adrenal 11β-hydroxylase, causing transient adrenal suppression that peaks early and largely resolves within 48 h after a single bolus dose [15]. Historical concerns about etomidate-associated mortality in sepsis and other critical illnesses have been tempered by four randomized controlled trials [16,17,18,19]—including the recent multicenter RSI trial by Casey et al. (NEJM 2026) [19]—demonstrating equivalent mortality outcomes compared to ketamine in general critically ill populations. Notably, while mortality is equivalent, ketamine has been associated with higher rates of cardiovascular collapse and post-intubation hypotension than etomidate in some studies [19,20,21].

These findings from general critically ill populations may not, however, translate to COVID-19 patients. The direct adrenal viral tropism, structural adrenal damage, inflammatory cell infiltration, and high prevalence of subclinical HPA-axis dysfunction in COVID-19 represent disease-specific pathophysiology that could render transient adrenal suppression clinically consequential in COVID-19 patients, even when it has no apparent effect in other critically ill patients. Limited observational evidence has suggested potentially worse outcomes with etomidate in COVID-19 patients. Edalatkhah et al. compared etomidate, ketamine, sodium thiopental, and midazolam in 76 COVID-19 patients; etomidate-treated patients had higher 7-day mortality (100%, *n* = 12) compared with the other agents (25–66%, *p* = 0.007) [22]. Leou et al. examined 171 COVID-19 patients across three countries and found higher 30-day mortality with etomidate versus other induction agents (80% vs. 59%, *p* = 0.006) [23]. Both studies, however, were limited by small sample sizes, heterogeneous comparators, potential confounding, and lack of propensity score matching.

We hypothesized that COVID-19 patients represent a uniquely vulnerable population in whom etomidate-induced adrenal suppression is associated with worse outcomes compared to ketamine. Using the TriNetX US Collaborative Network, we conducted a propensity-matched cohort study comparing 30-day mortality and vasopressor requirements between etomidate and ketamine in the largest cohort of COVID-19 patients requiring emergency intubation to date.

## 2. Materials and Methods

### 2.1. Study Design and Data Source

This retrospective cohort study utilized the TriNetX US Collaborative Network, a federated platform providing access to de-identified electronic health records from healthcare organizations across the United States [24,25]. The federated architecture enables real-time analysis while maintaining HIPAA and GDPR compliance without requiring data transfer between institutions [24]. At the time of analysis, the network contained over 110 million patient records [25] with comprehensive clinical data including demographics, diagnoses, procedures, medications, laboratory values, and vital signs. This study received approval from the Institutional Review Board of Shin Kong Wu Ho-Su Memorial Hospital (IRB number: 20250911R), with waiver of informed consent given the use of de-identified data.

### 2.2. Study Population

We identified adult patients (≥18 years) with COVID-19 infection who underwent emergency intubation with either etomidate or ketamine as the induction agent between 1 January 2020, and 30 June 2025. COVID-19 infection was identified by ICD-10 diagnosis code U07.1 or positive SARS-CoV-2 test within 14 days before or on the day of intubation. All patients had COVID-19 respiratory complications requiring mechanical ventilation and received a neuromuscular blocking agent (succinylcholine or rocuronium) at intubation. Complete query criteria for the etomidate and ketamine cohorts, including all ICD, CPT, and RxNorm codes, are provided in Supplementary Tables S1 and S2.

Patients were excluded if they: (1) received both etomidate and ketamine on the intubation day; (2) received the same induction agent within one day before or after index intubation, suggesting continuous sedation rather than single-dose induction; (3) had pre-existing adrenal insufficiency (within one year before intubation); (4) were pregnant (within three months before intubation); (5) had prior tracheostomy (within one year); or (6) underwent an anesthesia procedure on the intubation day, as this suggests elective rather than emergency intubation. For the primary cohort, patients with vasopressor use on day −1 were also excluded.

After applying these criteria, the initial cohort consisted of 67,910 patients (58,420 etomidate; 9490 ketamine). Following 1:1 propensity score matching, the final primary cohort comprised 9462 patients per group.

### 2.3. Exposure and Covariate Definitions

The index day (day 0) was defined as the calendar day of intubation with induction agent administration. Day −1 represents the calendar day immediately before the intubation day (day 0).

For baseline characterization and stratified analyses, vasopressor use was defined as administration of any of the following agents: epinephrine, norepinephrine, dopamine, vasopressin, or phenylephrine. This comprehensive definition was used to characterize hemodynamic status at baseline (day −1) and index day (day 0), as any vasopressor use—including epinephrine—indicates hemodynamic instability or cardiovascular compromise. For outcome assessment, epinephrine was excluded to focus on sustained hemodynamic support rather than resuscitation events.

Baseline covariates were assessed from one year prior through day 0 and included: (1) demographics (age, sex, race, ethnicity); (2) comorbidities (diabetes, obesity, hypertension, cardiovascular disease, cerebrovascular disease, chronic respiratory disease, liver disease, chronic kidney disease, malignancy); (3) medications (neuromuscular blocking agents, SARS-CoV-2 vaccine, corticosteroids, COVID-19 therapies); and (4) clinical and laboratory parameters (vital signs, body mass index, complete blood count, metabolic panel, liver function tests, coagulation studies, inflammatory markers, cardiac biomarkers, lactate). Complete covariate definitions, including all diagnostic codes and laboratory parameter specifications, are provided in Supplementary Table S3.

### 2.4. Propensity Score Matching

Propensity scores estimating the probability of receiving etomidate versus ketamine were derived using logistic regression incorporating all baseline covariates. One-to-one nearest-neighbor matching without replacement was performed using a caliper width of 0.1 pooled standard deviations. Covariate balance was assessed using standardized mean differences, with values < 0.10 considered adequate. Body temperature was categorized using a fever threshold of 100.4 °F (38.0 °C) to achieve adequate balance.

### 2.5. Outcomes

The primary outcome was all-cause mortality within 30 days of intubation.

Secondary outcomes included vasopressor requirements during days 0–30 and new diagnosis of adrenal insufficiency within 30 days. For the vasopressor outcome measure, epinephrine was excluded as it is primarily administered during cardiac arrest rather than for sustained hemodynamic support. Thus, vasopressor requirements (days 0–30) were defined as use of norepinephrine, dopamine, vasopressin, or phenylephrine, reflecting the need for ongoing hemodynamic support rather than resuscitation efforts. Adrenal insufficiency was defined by ICD-10 codes E27.1–E27.4 or serum cortisol ≤ 9.90 μg/dL (273 nmol/L). Complete diagnostic codes for all outcomes are provided in Supplementary Table S4.

Pre-specified sensitivity analyses included time-windowed mortality (days 0–7, 0–14, 0–21, and 0–30). Negative control outcomes (cystitis and cellulitis) were used to tested for residual baseline imbalance. Subgroup analyses examined consistency of the primary association across age, sex, body mass index, comorbidities, neuromuscular blocking agent, and pandemic period.

### 2.6. Stratified Analyses

To examine treatment effects across patients with different baseline hemodynamic status, we performed pre-specified stratified analyses by creating three independent propensity-matched cohorts using identical baseline covariates and matching methodology but different vasopressor inclusion criteria:

Primary matched cohort (*n* = 9462 pairs): Excluded patients with vasopressor on day −1; day 0 use not restricted.

Hemodynamically stable cohort (*n* = 2607 pairs): Excluded vasopressor use on day −1 or day 0, isolating delayed drug effects from peri-intubation confounding.

Critically ill cohort (*n* = 6557 pairs): Required vasopressor on both day −1 and day 0, examining effects in established shock.

### 2.7. Statistical Analysis

Baseline characteristics were summarized using means with standard deviations for continuous variables and frequencies with percentages for categorical variables. For time-to-event outcomes, Kaplan–MeierKaplan-Meier survival analysis was performed and hazard ratios with 95% confidence intervals were calculated using Cox proportional hazards models. All analyses were performed using the TriNetX Analytics platform with two-sided tests and significance level α = 0.05. All analyses were performed using the TriNetX Analytics platform (TriNetX, LLC., Cambridge, MA, USA) with two-sided tests and significance level α = 0.05. Figures were generated using R version 4.5.2 (R Foundation for Statistical Computing, Vienna, Austria) within RStudio version 2025.09.2 (Posit Software, PBC, Boston, MA, USA).

### 2.8. Use of Generative Artificial Intelligence

Generative artificial intelligence tool Claude Opus 4.5 (Anthropic, San Francisco, CA, USA) was used to assist with text drafting, language refinement, structural organization of the manuscript, and modification of R code for figure generation. The study design, data analysis, statistical interpretation, references, and scientific content were all the authors’ own work. The authors reviewed and edited all AI-assisted text and are responsible for the manuscript.

## 3. Results

### 3.1. Study Population and Baseline Characteristics

**Figure 1:**
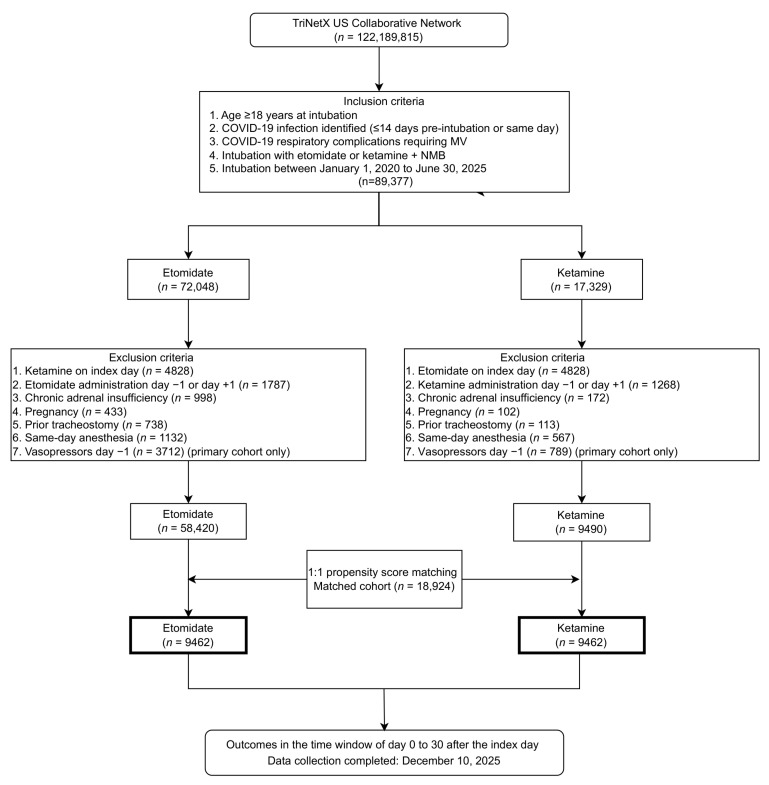
Patient selection flowchart for the primary matched cohort. Abbreviations: MV, mechanical ventilation; NMB, neuromuscular blockade.

Among 122,189,815 patients in the TriNetX US Collaborative Network, 89,377 adults met the inclusion criteria. After applying exclusion criteria, 67,910 patients (etomidate, *n* = 58,420; ketamine, *n* = 9490) remained eligible for matching. Following 1:1 propensity score matching, the final primary cohort comprised 9462 patients per group (Figure 1). Baseline characteristics were well balanced, with standardized mean differences <0.10 for all matched covariates (Table 1). The mean age was 58.2 years, and 40.7% were female.

### 3.2. Primary Outcome

**Figure 2:**
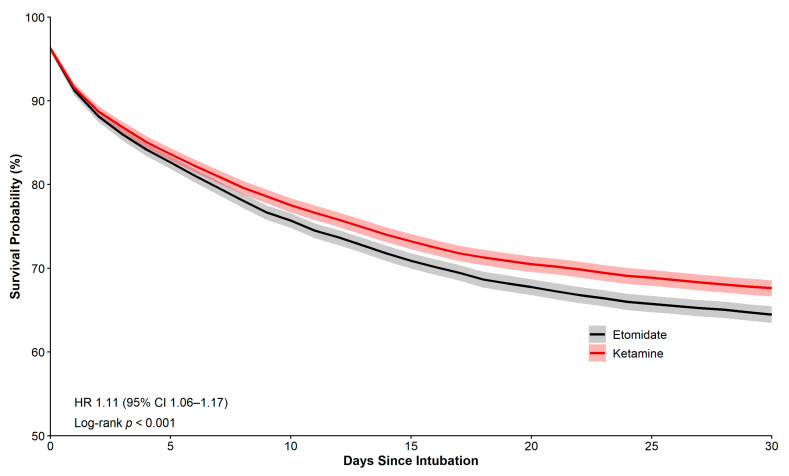
Thirty-Day Survival in Propensity-Matched Cohort.

Etomidate was associated with higher 30-day mortality than ketamine (3258 deaths [34.43%] vs. 2987 deaths [31.57%]; absolute risk difference 2.86 percentage points, number needed to harm (NNH) 35; HR 1.11, 95% CI 1.06–1.17, *p* < 0.001; Table 2). Kaplan–MeierKaplan-Meier curves separated progressively over follow-up (Figure 2).

Figure 2 shows Kaplan–Meier survival curves for propensity-matched COVID-19 patients requiring emergency intubation (*n* = 9462 per group). Etomidate (black line) and ketamine (red line) showed divergent survival with consistently lower survival in the etomidate group. Shaded areas (gray for etomidate, pink for ketamine) represent 95% confidence intervals. The hazard ratio was 1.11 (95% CI 1.06–1.17, log-rank *p* < 0.001).

### 3.3. Secondary Outcomes

Vasopressor use (excluding epinephrine) during days 0–30 was lower with etomidate (6537 [69.09%] vs. 7133 [75.39%]; HR 0.86, 95% CI 0.83–0.89, *p* < 0.001; Supplementary Figure S1). New diagnoses of adrenal insufficiency did not differ significantly between groups (213 [2.25%] vs. 240 [2.54%]; HR 0.89, 95% CI 0.74–1.07, *p* = 0.224).

### 3.4. Stratified Analyses by Hemodynamic Status

#### 3.4.1. Hemodynamically Stable Patients

**Figure 3:**
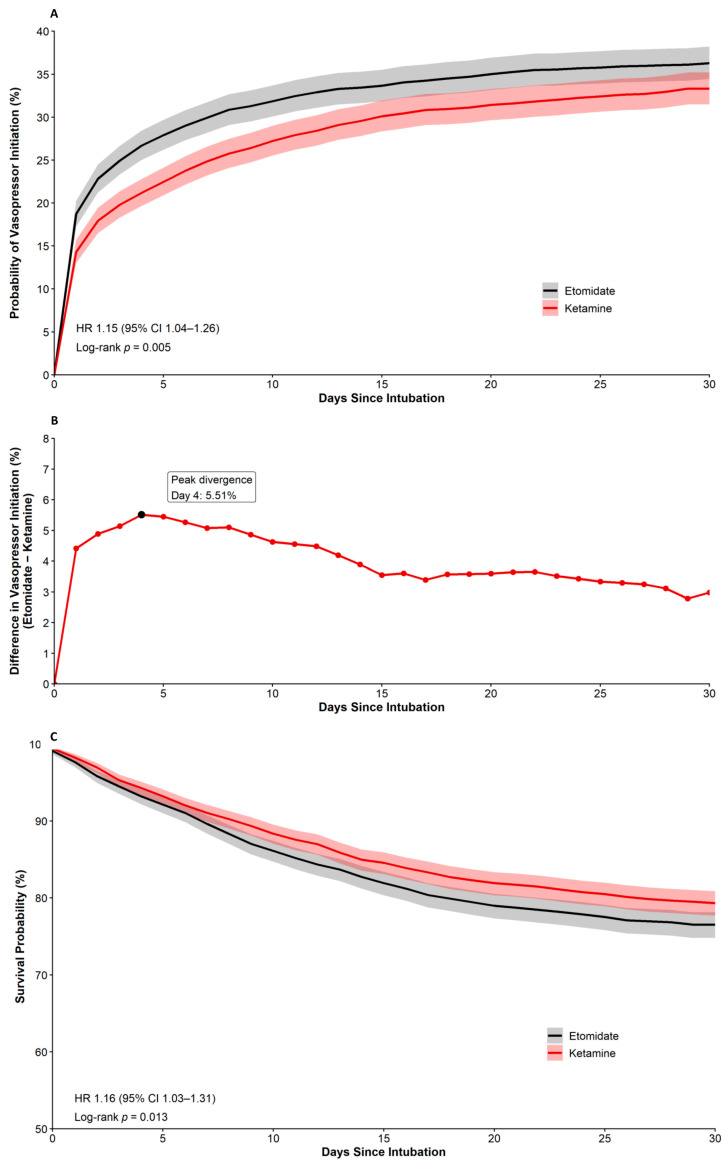
Vasopressor Initiation and 30-Day Survival in Hemodynamically Stable COVID-19 Patients, (**A**) Kaplan–Meier curves for cumulative incidence of vasopressor use; (**B**) differences in cumulative vasopressor incidence; (**C**) Kaplan–Meier curves for 30-day all-cause mortality.

Among 2607 matched pairs without vasopressor use from day −1 through day 0, etomidate was associated with higher 30-day mortality (582 [22.32%] vs. 520 [19.95%]; absolute risk difference 2.37 percentage points, number needed to harm 42; HR 1.16, 95% CI 1.03–1.31, *p* = 0.013). In this subgroup, in contrast to the primary cohort, etomidate was associated with higher vasopressor initiation during days 0–30 (914 [35.06%] vs. 836 [32.07%]; HR 1.15, 95% CI 1.04–1.26, *p* = 0.005; Figure 3A). The cumulative incidence difference (etomidate minus ketamine) was 4.41% at day 1, reached a peak of 5.51% at day 4, and declined below the day 1 level (to 4.41%) by day 13 (Figure 3B).

Figure 3 shows temporal patterns of vasopressor initiation and survival among patients without vasopressor use from day −1 through day 0 (*n* = 2607 per group). Figure 3A displays Kaplan–Meier curves showing cumulative incidence of vasopressor use. Etomidate (black line) and ketamine (red line) showed divergent early slopes. Shaded areas (gray for etomidate, pink for ketamine) represent 95% confidence intervals (HR 1.15, 95% CI 1.04–1.26, log-rank *p* = 0.005). Figure 3B shows that cumulative incidence difference (etomidate minus ketamine) peaked at day 4 (5.51%) and declined to below day 1 levels (4.41%) by day 13, demonstrating early divergence with subsequent narrowing. Figure 3C displays Kaplan–Meier survival curves showing 30-day mortality. Etomidate (black line) and ketamine (red line) curves were almost overlapping through day 1, separated gradually from around day 2–3, and continued to widen through day 30. Shaded areas (gray for etomidate, pink for ketamine) represent 95% confidence intervals (HR 1.16, 95% CI 1.03–1.31, log-rank *p* = 0.013).

#### 3.4.2. Critically Ill Patients with Persistent Vasopressor Requirements

Among 6557 matched pairs with vasopressor use on both day −1 and day 0, etomidate was associated with higher 30-day mortality (2813 [42.90%] vs. 2523 [38.48%]; absolute risk difference 4.42 percentage points, number needed to harm 23; HR 1.15, 95% CI 1.09–1.21, *p* < 0.001).

### 3.5. Sensitivity Analyses

Mortality hazard ratios rose progressively with longer follow-up windows (day 0–7: HR 1.05, 95% CI 0.98–1.12, *p* = 0.136; day 0–14: HR 1.08, 95% CI 1.02–1.14, *p* = 0.005; day 0–21: HR 1.10, 95% CI 1.04–1.15, *p* < 0.001; day 0–30: HR 1.11, 95% CI 1.06–1.17, *p* < 0.001; Table 2), consistent with a delayed rather than peri-intubation effect. Negative control outcomes showed no significant association (cystitis: HR 1.12, 95% CI 0.94–1.35, *p* = 0.200; cellulitis: HR 0.95, 95% CI 0.81–1.12, *p* = 0.560).

### 3.6. Subgroup Analyses

**Figure 4:**
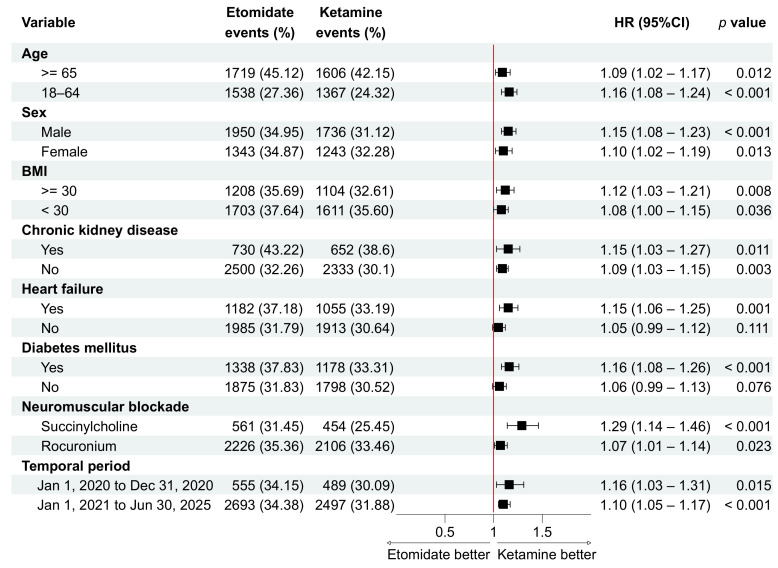
Subgroup analyses of 30-day mortality. Abbreviations: HR, hazard ratio; CI, confidence interval; BMI, body mass index.

The association between etomidate and 30-day mortality was directionally consistent across all prespecified subgroups (95% CIs and *p* values shown in Figure 4). Hazard ratios reached statistical significance across age groups (≥65 years: HR 1.09; 18–64 years: HR 1.16), sex (men: HR 1.15; women: HR 1.10), body mass index categories (≥30: HR 1.12; <30: HR 1.08), and in patients with or without chronic kidney disease (HR 1.15 and 1.09, respectively). The mortality association was stronger in certain comorbidity subgroups: among patients with heart failure, the HR was 1.15 (95% CI 1.06–1.25, *p* = 0.001) compared with HR 1.05 (95% CI 0.99–1.12, *p* = 0.111) in those without; among patients with diabetes mellitus, the HR was 1.16 (95% CI 1.08–1.26, *p* < 0.001) compared with HR 1.06 (95% CI 0.99–1.13, *p* = 0.076) in those without. The association was larger with succinylcholine (HR 1.29, 95% CI 1.14–1.46) than with rocuronium (HR 1.07, 95% CI 1.01–1.14). Results were consistent across the pandemic (2020: HR 1.16, 95% CI 1.03–1.31) and subsequent (2021–2025: HR 1.10, 95% CI 1.05–1.17) periods.

Figure 4 displays a forest plot showing hazard ratios and 95% confidence intervals for 30-day mortality associated with etomidate versus ketamine use across prespecified subgroups. Each subgroup analysis was performed using separate 1:1 propensity score matching within that subgroup.

## 4. Discussion

In this propensity-matched cohort study of 18,924 COVID-19 patients requiring emergency intubation, etomidate was associated with higher 30-day mortality compared with ketamine (HR 1.11, 95% CI 1.06–1.17), and this association persisted across the hemodynamic spectrum (HR 1.16 in stable patients; HR 1.15 in those with persistent vasopressor requirements). These findings stand in contrast to recent randomized trials in general critically ill populations [16,17,18,19] and raise the possibility that COVID-19 represents a disease-specific population in which etomidate-induced adrenal suppression carries clinically meaningful consequences.

### 4.1. Why COVID-19 May Differ from General Critical Illness

Four randomized trials—Jabre et al. (2009) [16], Matchett et al. (2022) [17], Knack et al. (2023) [18], and Casey et al. (2026) [19]—have uniformly reported no mortality difference between etomidate and ketamine in heterogeneous critically ill cohorts. These results should not, however, be uncritically extrapolated to COVID-19, given the disease-specific adrenal pathology and HPA-axis dysregulation described in the Introduction. A single bolus of etomidate inhibits 11β-hydroxylase: 80% of critically ill patients have measurable adrenal suppression at 12 h, falling to 9% by 48 h and 7% by 72 h [15]. In patients with intact adrenal reserve, this transient suppression appears clinically inconsequential. In patients whose adrenal reserve is already reduced by viral injury and subclinical HPA-axis compromise, however, the same insult may convert subclinical compromise into clinically meaningful adrenal insufficiency at the moment when stress-response cortisol is most needed.

### 4.2. Why Adrenal Suppression May Increase Mortality

The downstream effects of cortisol deficiency go beyond simple hormone replacement. Cortisol keeps blood vessels responsive to catecholamines—giving hydrocortisone to patients with septic shock restores the dose–response relationship to phenylephrine, showing that cortisol determines how well vasopressors work rather than acting as a vasopressor itself [26]. When cortisol is low, the same dose of vasopressor produces a smaller blood pressure response, so more vasopressor is needed. In addition, 11β-hydroxylase inhibition reduces both cortisol and aldosterone synthesis [27], and the loss of aldosterone may add a fluid-handling problem that pure glucocorticoid replacement does not address. Cortisol also helps restrain excessive inflammation; in critical illness this restraint can be impaired both by inadequate cortisol production and by reduced tissue responsiveness to cortisol [27]. In COVID-19, where unrestrained inflammation is a defining feature, even a brief drop in cortisol may allow inflammation to escape control, contributing to organ dysfunction that develops over days to weeks rather than to immediate cardiovascular collapse. Two distinct effects—reduced vasopressor responsiveness early, then disordered inflammation later—together fit the timing we observed. Consistent with this transient nature, in our cohort, the incidence of newly diagnosed adrenal insufficiency within 30 days did not differ significantly between groups (HR 0.89, 95% CI 0.74–1.07, *p* = 0.224). This is expected: routine clinical diagnosis of adrenal insufficiency requires sustained cortisol depression and clinical suspicion prompting ACTH or cortisol testing—neither of which would be triggered by the transient, often subclinical suppression that follows a single bolus of etomidate.

### 4.3. Different Timing of Vasopressor and Mortality Differences

In the hemodynamically stable subgroup (*n* = 2607 pairs), vasopressor and mortality differences appeared at different times. The vasopressor difference appeared early: most of the between-group difference was already present by the end of the first day (4.41%), reached a maximum at day 4 (5.51%), and then narrowed as ketamine-treated patients gradually accumulated vasopressor use, returning to 4.41% by day 13 (Figure 3B). The early vasopressor difference coincides with the window during which roughly 80% of critically ill patients receiving etomidate have measurable adrenal suppression [15]. By contrast, the mortality difference appeared late: Kaplan–Meier curves were almost overlapping through day 1, separated gradually from around day 2–3, and continued to widen throughout 30-day follow-up to reach a final mortality difference of 2.37 percentage points (Figure 3C). This dissociation—vasopressor difference closing by day 13 while mortality difference kept widening—argues against ongoing hemodynamic damage and supports a delayed mechanism in which transient peri-intubation 11β-hydroxylase inhibition initiates a cascade whose ultimate consequence shows up later.

The progressive widening of mortality is reinforced by time-windowed analysis in the primary matched cohort: hazard ratios rose progressively as the follow-up window lengthened (day 0–7 HR 1.05, *p* = 0.136; day 0–14 HR 1.08, *p* = 0.005; day 0–21 HR 1.10, *p* < 0.001; day 0–30 HR 1.11, *p* < 0.001). The day 0–7 difference was not statistically significant; the difference only reached significance from day 14 onward, again arguing against acute peri-intubation effects as the main mechanism.

### 4.4. Interpreting the Primary Cohort Findings

The primary cohort has one inherent limitation: we do not know the patients’ hemodynamic status at the moment of intubation. Because vasopressor use on day 0 was not restricted, TriNetX’s daily-resolution data cannot determine whether vasopressors were already being administered before intubation or were initiated afterward. This makes the primary cohort’s vasopressor outcome difficult to interpret.

In the primary cohort, vasopressor use was higher in the ketamine arm (75% vs. 69%) (Supplementary Figure S1). Most of this difference was already established by the end of day 0, with the gap subsequently narrowing rather than widening. Two explanations are possible. First, ketamine patients may have been sicker at the time of intubation, with more of them already on vasopressors. Second, ketamine itself may have caused more post-intubation hypotension, as reported in a recent randomized trial [19] and observational studies [20,21]. However, the first explanation fits our overall findings better. If ketamine were truly causing more post-intubation hypotension, the same pattern should appear in the hemodynamically stable cohort. Instead, among patients with no vasopressor use from day −1 through day 0, etomidate was associated with more subsequent vasopressor initiation (HR 1.15, *p* = 0.005). This opposite pattern argues against ketamine-induced hypotension as the explanation and instead suggests that ketamine patients in the primary cohort were more severely ill at baseline than the etomidate patients.

If this is correct, the mortality association observed in the primary cohort may actually be conservative. If ketamine patients were already sicker, they would have higher mortality regardless of induction agent—and this would shrink (not enlarge) the observed mortality gap favoring ketamine.

Our stratified analyses were designed to avoid this problem by examining patients with known hemodynamic status. The hemodynamically stable cohort (*n* = 2607 pairs) included only patients with no vasopressor use from day −1 through day 0—so we know both groups started intubation in similarly stable condition. The critically ill cohort (*n* = 6557 pairs) included only patients already on vasopressors on both day −1 and day 0—so we know both groups started intubation in similarly severe shock. In both cohorts, etomidate was associated with higher 30-day mortality (HR 1.16 and HR 1.15, respectively). The fact that the same finding appears in two opposite hemodynamic situations—both with known and balanced baseline conditions—argues that the mortality association is not just a reflection of one group being more critically ill than the other.

### 4.5. Clinical Orientation

As an observational study, our findings are hypothesis-generating and serve to inform clinical orientation rather than dictate practice. The absolute mortality difference was 2.86 percentage points (NNH 35) in the primary cohort. Although relative effect sizes were similar across hemodynamic strata (HR 1.11–1.16), absolute impact varied substantially with baseline risk: in stable patients the absolute difference was 2.37 percentage points (NNH 42), whereas in critically ill patients with established shock—where baseline mortality is highest—the absolute difference reached 4.42 percentage points (NNH 23). The clinical impact of induction agent choice is therefore amplified in the most severely ill COVID-19 patients. Although etomidate is conventionally valued for its cardiovascular stability during induction, and randomized trials in general populations report more peri-intubation cardiovascular instability with ketamine, our COVID-19 cohort showed the opposite pattern in the hemodynamically stable subgroup—where confounding by indication is minimized—with etomidate associated with greater vasopressor requirements. This suggests that etomidate’s anticipated cardiovascular advantage may not be realized in COVID-19 patients. For COVID-19 patients without contraindications to ketamine, our findings—taken together with prior observational literature [22,23]—suggest ketamine may be the preferred induction agent. The decision must be individualized: contraindications to ketamine, pre-intubation hemodynamic status, and clinical context all remain relevant, and observational evidence cannot displace the randomized data in general critical illness [16,17,18,19].

The comparative evidence among non-etomidate induction agents remains limited. In one COVID-19 study, 7-day mortality was highest with etomidate (100%) and lowest with ketamine (25%), although mortality with sodium thiopental (62%) and midazolam (66%) was also substantial [22]. In another COVID-19 study, etomidate was associated with higher mortality than a comparison group composed predominantly of propofol (74%), with smaller proportions receiving midazolam or ketamine [23]. These data suggest that ketamine—and possibly propofol—may be preferable to etomidate, whereas the relative safety of midazolam or sodium thiopental is less clear; no single alternative can be firmly recommended on the current evidence.

Whether this association can be mitigated by corticosteroid supplementation or by modification of the etomidate dose cannot be answered by our data. Current Society of Critical Care Medicine guidelines do not recommend routine corticosteroid administration to counteract etomidate-induced adrenal suppression following rapid sequence intubation, although this recommendation does not apply to patients already receiving corticosteroids or requiring them for other indications [28]. This position is consistent with randomized evidence, as hydrocortisone supplementation after single-dose etomidate did not improve mortality in critically ill patients without septic shock [29]. In COVID-19 specifically, a multicenter study found that stress-dose steroids did not significantly affect mortality after multivariable adjustment [23]. Furthermore, although the RECOVERY trial established dexamethasone as standard care for COVID-19 patients requiring respiratory support [30], the higher mortality with etomidate in our cohort persisted in both the pre-RECOVERY period (2020; HR 1.16) and the post-RECOVERY period (2021–2025; HR 1.10) in our temporal subgroup analysis. Together, these observations suggest that standard corticosteroid dosing does not fully offset the association. Whether higher, adrenal-replacement-level or pre-emptive corticosteroid administration specifically in COVID-19 patients could mitigate this effect remains untested and should be regarded as a hypothesis requiring prospective evaluation. Because the database does not capture administered doses, we could not assess whether dose modification of etomidate would alter outcomes.

Our findings do not establish an absolute contraindication to etomidate in COVID-19. Randomized evidence in general populations remains neutral, and a single observational study cannot justify a categorical prohibition. Rather, our results suggest that clinicians may reasonably consider an alternative agent such as ketamine in appropriate patients, maintain vigilance for adrenal insufficiency, and individualize decisions according to the clinical context. A randomized trial restricted to COVID-19 would directly test this hypothesis.

### 4.6. Strengths and Limitations

Strengths include the large sample size (*n* = 18,924), rigorous propensity score matching, pre-specified stratified analyses examining patients with known baseline hemodynamic status, sensitivity analyses across multiple time windows, and negative control outcomes.

Limitations include: (1) observational data cannot establish causation; unmeasured confounders—particularly bedside clinical judgment at the time of intubation—may influence results; (2) TriNetX captures events at daily resolution, precluding determination of whether day 0 vasopressor administration occurred before or after intubation; (3) HPA-axis function (serial cortisol, ACTH stimulation) could not be directly measured to confirm the proposed mechanism; (4) although TriNetX records the medication product and its formulation strength (e.g., 2 mg/mL), it does not capture the administered dose; the etomidate and ketamine doses therefore could not be verified at the patient level. In addition, subsequent corticosteroid therapy was not assessed or adjusted for and represents a potential unmeasured confounder, whereas baseline corticosteroid exposure was balanced through propensity score matching.; (5) the prevalence of subclinical adrenal insufficiency was inferred from the literature [8,10] rather than directly measured; and (6) the study included only COVID-19 patients; the apparent disease-specific effect rests on indirect comparison with randomized trials in general critically ill populations rather than a head-to-head non-COVID comparison.

## 5. Conclusions

In this large propensity-matched cohort study, etomidate was associated with higher 30-day mortality than ketamine in COVID-19 patients across the hemodynamic spectrum, unlike the results seen in general critical illness. This supports disease-specific adrenal vulnerability. Ketamine may be the preferred induction agent in COVID-19 patients without contraindications, pending randomized validation.

## Figures and Tables

**Table 1:** Baseline Characteristics of the Study Population Before and After Propensity Score Matching.

	Before Matching			After Matching		
Variable	Etomidate	Ketamine		Etomidate	Ketamine	
	(*n* = 58,420)	(*n* = 9490)	SMD	(*n* = 9462)	(*n* = 9462)	SMD
Age at Index (years)	60.9 ± 15.8	58.1 ± 16.5	0.175	58.3 ± 16.6	58.1 ± 16.5	0.012
Male, *n* (%)	33,873 (58.0)	5623 (59.3)	0.026	5624 (59.4)	5605 (59.2)	0.004
Female, *n* (%)	24,522 (42.0)	3866 (40.7)	0.025	3836 (40.5)	3856 (40.8)	0.004
Race, *n* (%)						
White	36,701 (62.8)	6005 (63.3)	0.009	5902 (62.4)	5987 (63.3)	0.019
African American	12,688 (21.7)	2073 (21.8)	0.003	2177 (23.0)	2069 (21.9)	0.027
Asian	2796 (4.8)	243 (2.6)	0.119	267 (2.8)	243 (2.6)	0.016
Other Race	2696 (4.6)	351 (3.7)	0.046	327 (3.5)	351 (3.7)	0.014
Unknown Race	2185 (3.7)	453 (4.8)	0.051	435 (4.6)	451 (4.8)	0.008
Ethnicity, *n* (%)						
Not Hispanic or Latino	46,845 (80.2)	7243 (76.3)	0.094	7279 (76.9)	7223 (76.3)	0.014
Hispanic or Latino	5118 (8.8)	1095 (11.5)	0.092	1061 (11.2)	1088 (11.5)	0.009
Unknown Ethnicity	6457 (11.1)	1152 (12.1)	0.034	1122 (11.9)	1151 (12.2)	0.009
**Comorbidity, *n* (%)**						
Diabetes mellitus	24,006 (41.1)	3543 (37.3)	0.077	3517 (37.2)	3539 (37.4)	0.005
Overweight/obesity	18,964 (32.5)	2891 (30.5)	0.043	2927 (30.9)	2885 (30.5)	0.010
Hypertensive diseases	43,368 (74.2)	6370 (67.1)	0.157	6321 (66.8)	6365 (67.3)	0.010
Ischemic heart diseases	25,880 (44.3)	3606 (38.0)	0.128	3679 (38.9)	3604 (38.1)	0.016
Atrial fibrillation/flutter	17,338 (29.7)	2425 (25.6)	0.092	2485 (26.3)	2423 (25.6)	0.015
Heart failure	22,900 (39.2)	3188 (33.6)	0.117	3240 (34.2)	3187 (33.7)	0.012
Cerebrovascular diseases	16,791 (28.7)	2060 (21.7)	0.163	2068 (21.9)	2059 (21.8)	0.002
Chronic lower respiratory diseases	21,475 (36.8)	3663 (38.6)	0.038	3750 (39.6)	3654 (38.6)	0.021
Hepatic failure	6888 (11.8)	1310 (13.8)	0.060	1313 (13.9)	1307 (13.8)	0.002
Liver cirrhosis	4202 (7.2)	840 (8.9)	0.061	841 (8.9)	836 (8.8)	0.002
Alcoholic liver disease	2994 (5.1)	685 (7.2)	0.087	650 (6.9)	681 (7.2)	0.013
CKD, stage 3	8726 (14.9)	1189 (12.5)	0.070	1215 (12.8)	1188 (12.6)	0.009
CKD, stage 4	2915 (5.0)	413 (4.4)	0.030	435 (4.6)	413 (4.4)	0.011
CKD, stage 5	1123 (1.9)	154 (1.6)	0.023	154 (1.6)	154 (1.6)	<0.001
ESRD	4450 (7.6)	625 (6.6)	0.040	612 (6.5)	625 (6.6)	0.006
Intracranial injury	4569 (7.8)	806 (8.5)	0.025	759 (8.0)	803 (8.5)	0.017
Malignancies, *n* (%)						
Central nerve system	305 (0.5)	41 (0.4)	0.013	37 (0.4)	41 (0.4)	0.007
Head/neck	272 (0.5)	70 (0.7)	0.035	81 (0.9)	70 (0.7)	0.013
Respiratory/thoracic	2072 (3.5)	344 (3.6)	0.004	339 (3.6)	343 (3.6)	0.002
Gastrointestinal	1644 (2.8)	339 (3.6)	0.043	345 (3.6)	336 (3.6)	0.005
Urinary tract	718 (1.2)	112 (1.2)	0.004	113 (1.2)	112 (1.2)	0.001
Urologic, male	884 (1.5)	150 (1.6)	0.005	126 (1.3)	150 (1.6)	0.021
Gynecologic	387 (0.7)	78 (0.8)	0.019	69 (0.7)	77 (0.8)	0.010
Breast	886 (1.5)	129 (1.4)	0.013	140 (1.5)	129 (1.4)	0.010
Hematologic	2310 (4.0)	340 (3.6)	0.020	329 (3.5)	340 (3.6)	0.006
Bone/cartilage	122 (0.2)	20 (0.2)	<0.001	15 (0.2)	20 (0.2)	0.012
Skin/melanoma	789 (1.4)	129 (1.4)	0.001	131 (1.4)	129 (1.4)	0.002
Mesothelioma/soft tissue	269 (0.5)	50 (0.5)	0.009	52 (0.6)	50 (0.5)	0.003
Neuroendocrine	162 (0.3)	34 (0.4)	0.014	28 (0.3)	34 (0.4)	0.011
Thyroid/other endocrine	148 (0.3)	29 (0.3)	0.010	31 (0.3)	29 (0.3)	0.004
Other/unspecified	3205 (5.5)	554 (5.8)	0.015	521 (5.5)	553 (5.8)	0.015
**Medication, *n* (%)**						
Succinylcholine	20,410 (34.9)	2843 (30.0)	0.106	2899 (30.6)	2841 (30.0)	0.013
Rocuronium	44,682 (76.5)	7820 (82.4)	0.147	7755 (82.0)	7793 (82.4)	0.010
SARS-CoV-2 vaccine	5498 (9.4)	734 (7.7)	0.060	750 (7.9)	734 (7.8)	0.006
Dexamethasone	19,174 (32.8)	3041 (32.0)	0.017	3015 (31.9)	3037 (32.1)	0.005
Prednisolone	964 (1.7)	161 (1.7)	0.004	143 (1.5)	160 (1.7)	0.014
Remdesivir	6619 (11.3)	783 (8.3)	0.104	793 (8.4)	783 (8.3)	0.004
Baricitinib	1224 (2.1)	142 (1.5)	0.045	139 (1.5)	142 (1.5)	0.003
Tocilizumab	1248 (2.1)	112 (1.2)	0.075	100 (1.1)	112 (1.2)	0.012
Nirmatrelvir	298 (0.5)	54 (0.6)	0.008	47 (0.5)	54 (0.6)	0.010
Ritonavir	280 (0.5)	37 (0.4)	0.014	24 (0.3)	37 (0.4)	0.024
Molnupiravir	88 (0.2)	14 (0.1)	0.001	13 (0.1)	14 (0.1)	0.003
**Clinical parameters, mean ± SD**						
BMI (kg/m^2^)	29.2 ± 8.6	29.7 ± 9.2	0.049	29.2 ± 8.7	29.7 ± 9.2	0.054
RR (breaths/min)	19.7 ± 6.0	20.2 ± 6.0	0.073	19.7 ± 6.0	20.2 ± 6.0	0.073
Heart rate (beats/min)	88.7 ± 23.6	89.2 ± 22.8	0.022	89.3 ± 23.7	89.3 ± 22.8	0.003
Oxygen saturation (%)	80.0 ± 25.6	77.4 ± 26.7	0.098	79.2 ± 26.5	77.4 ± 26.7	0.067
SBP (mmHg)	114.7 ± 27.1	113.3 ± 25.6	0.050	113.8 ± 26.5	113.4 ± 25.5	0.018
DBP (mmHg)	65.7 ± 17.7	65.4 ± 17.2	0.019	65.8 ± 17.6	65.4 ± 17.2	0.025
Body temperature (°F)						
≧100.4, *n* (%)	12,316 (21.1)	1686 (17.8)	0.084	1654 (17.5)	1682 (17.8)	0.008
<100.4, *n* (%)	46,290 (79.2)	7474 (78.8)	0.012	7379 (78.0)	7447 (78.7)	0.017
**Laboratory tests, mean ± SD**						
Hemoglobin (g/dL)	11.6 ± 3.0	11.5 ± 3.0	0.010	11.6 ± 3.0	11.5 ± 3.0	0.025
Leukocytes (×10^3^/uL)	14.2 ± 70.8	12.8 ± 10.7	0.027	15.4 ± 104.9	12.8 ± 10.7	0.035
Platelets (×10^3^/uL)	225.7 ± 118.7	224.0 ± 121.7	0.015	225.0 ± 121.1	224.0 ± 121.7	0.008
Neutrophils (×10^3^/uL)	12.7 ± 212.3	9.9 ± 7.3	0.019	13.0 ± 132.9	9.9 ± 7.3	0.033
Lymphocytes (%)	13.0 ± 12.7	13.3 ± 13.0	0.025	13.3 ± 12.7	13.3 ± 13.0	0.006
Urea nitrogen (mg/dL)	30.4 ± 24.5	29.9 ± 24.9	0.020	29.5 ± 24.7	29.9 ± 24.9	0.015
Creatinine (mg/dL)	1.7 ± 2.0	1.7 ± 2.3	0.008	1.7 ± 1.8	1.7 ± 2.3	0.009
Sodium (mmol/L)	137.2 ± 6.2	137.0 ± 6.3	0.018	137.1 ± 6.1	137.0 ± 6.3	0.015
Potassium (mmol/L)	4.0 ± 0.7	4.0 ± 0.8	<0.001	4.0 ± 0.7	4.0 ± 0.8	0.026
Bicarbonate (mmol/L)	21.2 ± 6.5	21.0 ± 6.7	0.035	21.2 ± 6.6	21.0 ± 6.7	0.032
Glucose (mg/dL)	140.9 ± 83.0	138.9 ± 81.6	0.024	137.6 ± 84.8	138.9 ± 81.7	0.016
ALT (U/L)	73.6 ± 272.0	91.2 ± 372.6	0.054	78.8 ± 283.5	91.0 ± 372.3	0.037
AST (U/L)	110.9 ± 447.2	147.0 ± 713.6	0.061	124.8 ± 489.8	146.4 ± 710.4	0.035
Total bilirubin (mg/dL)	1.2 ± 2.6	1.4 ± 3.4	0.073	1.4 ± 3.1	1.4 ± 3.4	0.004
Direct bilirubin (mg/dL)	0.9 ± 2.3	1.1 ± 3.3	0.099	1.0 ± 2.6	1.1 ± 3.3	0.062
Prothrombin time (s)	15.6 ± 6.7	16.1 ± 8.4	0.062	15.9 ± 6.9	16.1 ± 8.3	0.023
aPTT (s)	34.5 ± 16.1	35.0 ± 16.2	0.029	34.9 ± 16.9	35.0 ± 16.2	0.007
D-dimer FEU (mg/L)	357.4 ± 1511.0	270.6 ± 1314.9	0.061	291.6 ± 1397.3	271.7 ± 1317.7	0.015
CRP (mg/dL)	90.2 ± 95.9	90.8 ± 96.3	0.006	89.5 ± 97.4	90.8 ± 96.3	0.013
Procalcitonin (ng/mL)	5.5 ± 25.6	6.9 ± 28.8	0.048	5.1 ± 21.4	6.9 ± 28.8	0.069
ESR (mm/h)	45.1 ± 32.6	45.4 ± 32.7	0.011	45.3 ± 33.2	45.4 ± 32.7	0.004
Troponin I (ng/mL)	0.9 ± 9.2	0.7 ± 5.9	0.019	0.9 ± 7.9	0.7 ± 5.9	0.018
BNP (pg/mL)	1152.0 ± 3812.3	924.4 ± 3089.6	0.066	1051.7 ± 3496.0	924.4 ± 3089.6	0.039
Lactate (mmol/L)	2.7 ± 2.7	3.0 ± 3.4	0.122	2.8 ± 2.9	3.0 ± 3.4	0.092

Abbreviations: SMD, standardized mean difference; SD, standard deviation; CKD, chronic kidney disease; ESRD, end stage renal disease; BMI, body mass index; RR, respiratory rate; SBP, systolic blood pressure; DBP, diastolic blood pressure; ALT, alanine aminotransferase; AST, aspartate aminotransferase; aPTT, activated partial thromboplastin time; FEU, fibrinogen equivalent units; CRP, C reactive protein; ESR, erythrocyte sedimentation rate; BNP, B-type natriuretic peptide.

**Table 2:** Clinical Outcomes and Sensitivity Analyses After Propensity Score Matching.

Clinical Outcomes	After Matching						
		Etomidate		Ketamine		Effect Estimate	*p* Value
	*n*	Events	(%)	Events	(%)	(95% CI)	
Primary Outcome							
Mortality							
Day 0–30	9462	3258	34.43	2987	31.57	1.11 (1.06,1.17)	<0.001
Secondary Outcomes							
Vasopressors (excl. epinephrine)							
Day 0–30	9462	6537	69.09	7133	75.39	0.86 (0.83,0.89)	<0.001
Adrenal Insufficiency							
Day 0–30	9462	213	2.25	240	2.54	0.89 (0.74,1.07)	0.224
Stratified Analyses by Hemodynamic Status ^†^							
Hemodynamically stable patients (no vasopressor Day −1 to Day 0), Outcome Day 0–30							
Mortality	2607	582	22.32	520	19.95	1.16 (1.03,1.31)	0.013
Vasopressor use	2607	914	35.06	836	32.07	1.15 (1.04,1.26)	0.005
Critically ill patients (persistent vasopressor Day −1 and Day 0), Outcome Day 0–30							
Mortality	6557	2813	42.90	2523	38.48	1.15 (1.09,1.21)	<0.001
Sensitivity Analyses							
Mortality by time window ^‡^							
Mortality, Day 0–7	9462	1876	19.83	1794	18.96	1.05 (0.98,1.12)	0.136
Mortality, Day 0–14	9462	2597	27.45	2426	25.64	1.08 (1.02,1.14)	0.005
Mortality, Day 0–21	9462	2979	31.48	2761	29.18	1.10 (1.04,1.15)	<0.001
Negative Control (Day 0–30: Unrelated diagnoses) ^§^							
Cystitis	9462	252	2.66	226	2.39	1.12 (0.94,1.35)	0.200
Cellulitis	9462	271	2.86	286	3.02	0.95 (0.81,1.12)	0.560

Abbreviations: CI, confidence interval; Hazard ratio for time-to-event outcomes. ^†^ Stratified analyses by baseline hemodynamic status were pre-specified to examine treatment effects in patients with known hemodynamic status at the time of intubation. Hemodynamically stable patients had no vasopressor requirements from day −1 through day 0 (the peri-intubation period), representing patients with known stable hemodynamic status at intubation. Critically ill patients had persistent vasopressor requirements on both day −1 and day 0, representing patients with known established shock at intubation. ^‡^ Mortality by time window: Mortality assessed across different time windows should demonstrate consistent effect sizes if the association is real. ^§^ Negative control: Outcomes unrelated to drug mechanism should show no association, validating the specificity of findings.

## Data Availability

The data that support the findings of this study are available from TriNetX (TriNetX US Collaborative Network, www.trinetx.com) but restrictions apply to the availability of these data, which were used under license for the current study, and so are not publicly available. Data are however available from the authors upon reasonable request and with permission of TriNetX.

## Supplementary Materials

The following supporting information can be downloaded at: https://www.mdpi.com/article/10.3390/jcm15135050/s1, Figure S1: Vasopressor initiation in the primary matched cohort; Table S1: Query criteria for the etomidate cohort; Table S2: Query criteria for the ketamine cohort; Table S3: Propensity score matching parameters and covariates; Table S4: Outcome definitions and diagnostic codes.

## Abbreviations

The following abbreviations are used in this manuscript:

BMI	Body mass index
CI	Confidence interval
COVID-19	Coronavirus disease 2019
GenAI	Generative artificial intelligence
GDPR	General Data Protection Regulation
HIPAA	Health Insurance Portability and Accountability Act
HPA	Hypothalamic–pituitary–adrenal
HR	Hazard ratio
ICD-10	International Classification of Diseases, 10th Revision ICU Intensive care unit
IRB	Institutional Review Board
KM	Kaplan–Meier
NMB	Neuromuscular blocking agent
NNH	Number needed to harm
RSI	Rapid sequence intubation
SARS-CoV-2	Severe acute respiratory syndrome coronavirus 2
SMD	Standardized mean difference
