# Supplementary material for: Etomidate Is Associated with Higher 30-Day Mortality than Ketamine for Emergency Intubation of COVID-19 Patients: A Propensity-Matched Cohort Study
Source: J Clin Med. 2026 Jun 29;15(13):5050. doi: 10.3390/jcm15135050 (PMC13363312; doi:10.3390/jcm15135050)
Supplement: Supplementary file 1 [file jcm-15-05050-s001.zip › jcm-4347350-supplementary.pdf]

**Supplementary Methods. Detailed query criteria and analytical procedures.**

**Supplementary Tables:**

- Supplementary Table S1. Query criteria for the etomidate cohort.
- Supplementary Table S2. Query criteria for the ketamine cohort.
- Supplementary Table S3. Propensity score matching parameters and covariates.
- Supplementary Table S4. Outcome definitions and diagnostic codes.

**Supplementary Figures:**

- Vasopressor initiation in the primary matched cohort.

**Supplementary Table S1. Query Criteria for Cohort 1 (query name: Etomidate)**

This query was run on the network US Collaborative Network with 69 HCO(s) queried and 69 HCO(s) responded. A total of 56 provider(s) responded with patients.

| | | | | |
|---|---|---|---|---|
| **Group 1** | | | | |
| **Intubation** | | | | |
| must have | any of | procedure | UMLS:CPT:31500 | Intubation, endotracheal, emergency procedure (at least 18 years old at event) |
| | | procedure | UMLS:ICD10PCS:0BH17EZ | Insertion of Endotracheal Airway into Trachea, Via Natural or Artificial Opening (at least 18 years old at event) |
| date constraint | | The terms in this group occurred between Jan 1, 2020 and Jun 30, 2025 | | |
| **Group 2** | | | | |
| **Group 2A Intubation** | | | | |
| must have | any of | procedure | UMLS:CPT:31500 | Intubation, endotracheal, emergency procedure (at least 18 years old at event) |
| | | procedure | UMLS:ICD10PCS:0BH17EZ | Insertion of Endotracheal Airway into Trachea, Via Natural or Artificial Opening (at least 18 years old at event) |
| date constraint | | The terms in this group occurred between Jan 1, 2020 and Jun 30, 2025 | | |
| event relationship | | Any instance of confirmed COVID-19 infection occurred within 14 days on or before the first instance of Intubation | | |
| **Group 2B Confirmed COVID-19 infection** | | | | |
| must have | any of | diagnosis | UMLS:ICD10CM:U07.1 | COVID-19 |
| | | laboratory | TNX:9088 | SARS coronavirus 2 and related RNA [Presence] |
| | | laboratory | UMLS:LNC:94500-6 | SARS-CoV-2 (COVID-19) RNA [Presence] in Respiratory system specimen by NAA with probe detection |
| | | laboratory | UMLS:LNC:94502-2 | SARS-related coronavirus RNA [Presence] in Respiratory system specimen by NAA with probe detection |
| | | laboratory | UMLS:LNC:94565-9 | SARS-CoV-2 (COVID-19) RNA [Presence] in Nasopharynx by NAA with non-probe detection |
| | | laboratory | UMLS:LNC:95209-3 | SARS-CoV+SARS-CoV-2 (COVID-19) Ag [Presence] in Respiratory system specimen by Rapid immunoassay |
| | | laboratory | UMLS:LNC:96119-3 | SARS-CoV-2 (COVID-19) Ag [Presence] in Upper respiratory specimen by Immunoassay |

| | | | | |
|---|---|---|---|---|
| | | laboratory | UMLS:LNC:94309-2 | SARS-CoV-2 (COVID-19) RNA [Presence] in Specimen by NAA with probe detection |
| | | laboratory | UMLS:LNC:94306-8 | SARS-CoV-2 (COVID-19) RNA panel - Specimen by NAA with probe detection |
| | | laboratory | UMLS:LNC:94558-4 | SARS-CoV-2 (COVID-19) Ag [Presence] in Respiratory system specimen by Rapid immunoassay |
| | | laboratory | UMLS:LNC:94759-8 | SARS-CoV-2 (COVID-19) RNA [Presence] in Nasopharynx by NAA with probe detection |
| | | laboratory | UMLS:LNC:94845-5 | SARS-CoV-2 (COVID-19) RNA [Presence] in Saliva (oral fluid) by NAA with probe detection |
| | | laboratory | UMLS:LNC:95406-5 | SARS-CoV-2 (COVID-19) RNA [Presence] in Nose by NAA with probe detection |
| | | laboratory | UMLS:LNC:97097-0 | SARS-CoV-2 (COVID-19) Ag [Presence] in Upper respiratory specimen by Rapid immunoassay |
| **Group 3** | | | | |
| **Group 3A Intubation** | | | | |
| must have | any of | procedure | UMLS:CPT:31500 | Intubation, endotracheal, emergency procedure (at least 18 years old at event) |
| | | procedure | UMLS:ICD10PCS:0BH17EZ | Insertion of Endotracheal Airway into Trachea, Via Natural or Artificial Opening (at least 18 years old at event) |
| date constraint | | The terms in this group occurred between Jan 1, 2020 and Jun 30, 2025 | | |
| event relationship | | Any instance of COVID-19 respiratory complications occurred within 14 days on or before the first instance of Intubation | | |
| **Group 3B COVID-19 respiratory complications** | | | | |
| must have | any of | diagnosis | UMLS:ICD10CM:J12.82 | Pneumonia due to coronavirus disease 2019 |
| | | diagnosis | UMLS:ICD10CM:J12.9 | Viral pneumonia, unspecified |
| | | diagnosis | UMLS:ICD10CM:J80 | Acute respiratory distress syndrome |
| | | diagnosis | UMLS:ICD10CM:J96.0 | Acute respiratory failure |
| **Group 4** | | | | |
| **Group 4A Intubation** | | | | |
| must have | any of | procedure | UMLS:CPT:31500 | Intubation, endotracheal, emergency procedure (at least 18 years old at event) |
| | | procedure | UMLS:ICD10PCS:0BH17EZ | Insertion of Endotracheal Airway into Trachea, Via Natural or Artificial Opening (at least 18 years old at event) |
| date constraint | | The terms in this group occurred between Jan 1, 2020 and Jun 30, 2025 | | |
| event relationship | | Any instance of NMB occurred on the same date as the first instance of Intubation | | |
| **Group 4B NMB** | | | | |
| must have | any of | medication | NLM:RXNORM:10154 | succinylcholine |
| | | medication | NLM:RXNORM:68139 | rocuronium |
| **Group 5** | | | | |

| | | | | |
|---|---|---|---|---|
| **Group 5A Intubation** | | | | |
| must have | any of | procedure | UMLS:CPT:31500 | Intubation, endotracheal, emergency procedure (at least 18 years old at event) |
| | | procedure | UMLS:ICD10PCS:0BH17EZ | Insertion of Endotracheal Airway into Trachea, Via Natural or Artificial Opening (at least 18 years old at event) |
| date constraint | | The terms in this group occurred between Jan 1, 2020 and Jun 30, 2025 | | |
| event relationship | | Any instance of etomidate occurred on the same date as the first instance of Intubation | | |
| **Group 5B Etomidate** | | | | |
| must have | | medication | NLM:RXNORM:4177 | etomidate |
| **Group 6** | | | | |
| **Group 6A Intubation** | | | | |
| must have | any of | procedure | UMLS:CPT:31500 | Intubation, endotracheal, emergency procedure (at least 18 years old at event) |
| | | procedure | UMLS:ICD10PCS:0BH17EZ | Insertion of Endotracheal Airway into Trachea, Via Natural or Artificial Opening (at least 18 years old at event) |
| date constraint | | The terms in this group occurred between Jan 1, 2020 and Jun 30, 2025 | | |
| event relationship | | Any instance of exclude ketamine occurred on the same date as the first instance of Intubation | | |
| **Group 6B Exclude ketamine** | | | | |
| cannot have | | medication | NLM:RXNORM:6130 | ketamine |
| **Group 7** | | | | |
| **Group 7A Intubation** | | | | |
| must have | any of | procedure | UMLS:CPT:31500 | Intubation, endotracheal, emergency procedure (at least 18 years old at event) |
| | | procedure | UMLS:ICD10PCS:0BH17EZ | Insertion of Endotracheal Airway into Trachea, Via Natural or Artificial Opening (at least 18 years old at event) |
| date constraint | | The terms in this group occurred between Jan 1, 2020 and Jun 30, 2025 | | |
| event relationship | | Any instance of exclude etomidate administration after occurred exactly 1 day after the first instance of Intubation | | |
| **Group 7B Exclude etomidate administration after the index day** | | | | |
| cannot have | | medication | NLM:RXNORM:4177 | etomidate |
| **Group 8** | | | | |
| **Group 8A Intubation** | | | | |
| must have | any of | procedure | UMLS:CPT:31500 | Intubation, endotracheal, emergency procedure (at least 18 years old at event) |
| | | procedure | UMLS:ICD10PCS:0BH17EZ | Insertion of Endotracheal Airway into Trachea, Via Natural or Artificial Opening (at least 18 years old at event) |
| date constraint | | The terms in this group occurred between Jan 1, 2020 and Jun 30, 2025 | | |
| event relationship | | Any instance of exclude etomidate administration before occurred exactly 1 day before the first instance of Intubation | | |

| | | | | | |
|---|---|---|---|---|---|
| | **Group 8B Exclude etomidate administration before the index day** | | | | |
| | cannot have | | medication | NLM:RXNORM:4177 | etomidate |
| **Group 9** | | | | | |
| | **Group 9A Intubation** | | | | |
| | must have | any of | procedure | UMLS:CPT:31500 | Intubation, endotracheal, emergency procedure (at least 18 years old at event) |
| | | | procedure | UMLS:ICD10PCS:0BH 17EZ | Insertion of Endotracheal Airway into Trachea, Via Natural or Artificial Opening (at least 18 years old at event) |
| | date constraint | | The terms in this group occurred between Jan 1, 2020 and Jun 30, 2025 | | |
| | event relationship | | Any instance of no pregnancy occurred within 3 months on or before the first instance of Intubation | | |
| | **Group 9B No pregnancy** | | | | |
| | cannot have | | procedure | UMLS:ICD10PCS:10 | Pregnancy |
| | | or | diagnosis | UMLS:ICD10CM:O00-O9A | Pregnancy, childbirth and the puerperium |
| | | or | diagnosis | UMLS:ICD10CM:Z33.1 | Pregnant state, incidental |
| | | or | diagnosis | UMLS:ICD10CM:Z34 | Encounter for supervision of normal pregnancy |
| | | or | diagnosis | UMLS:ICD10CM:Z3A | Weeks of gestation |
| **Group 10** | | | | | |
| | **Group 10A Intubation** | | | | |
| | must have | any of | procedure | UMLS:CPT:31500 | Intubation, endotracheal, emergency procedure (at least 18 years old at event) |
| | | | procedure | UMLS:ICD10PCS:0BH 17EZ | Insertion of Endotracheal Airway into Trachea, Via Natural or Artificial Opening (at least 18 years old at event) |
| | date constraint | | The terms in this group occurred between Jan 1, 2020 and Jun 30, 2025 | | |
| | event relationship | | Any instance of anesthesia occurred on the same date as the first instance of Intubation | | |
| | **Group 10B Anesthesia** | | | | |
| | cannot have | | procedure | UMLS:CPT:1002796 | Anesthesia |
| **Group 11** | | | | | |
| | **Group 11A Intubation** | | | | |
| | must have | any of | procedure | UMLS:CPT:31500 | Intubation, endotracheal, emergency procedure (at least 18 years old at event) |
| | | | procedure | UMLS:ICD10PCS:0BH 17EZ | Insertion of Endotracheal Airway into Trachea, Via Natural or Artificial Opening (at least 18 years old at event) |
| | date constraint | | The terms in this group occurred between Jan 1, 2020 and Jun 30, 2025 | | |
| | event relationship | | Any instance of exclude previous tracheostomy occurred within 1 year and 1 day before the first instance of Intubation | | |
| | **Group 11B Exclude previous tracheostomy** | | | | |
| | cannot have | | diagnosis | UMLS:ICD10CM:Z93.0 | Tracheostomy status |

| | | | | |
|---|---|---|---|---|
| | or | procedure | UMLS:CPT:31600 | Tracheostomy, planned (separate procedure) |
| | or | procedure | UMLS:CPT:31603 | Tracheostomy, emergency procedure; transtracheal |
| | or | procedure | UMLS:CPT:31610 | Tracheostomy, fenestration procedure with skin flaps |
| **Group 12** | | | | |
| **Group 12A Intubation** | | | | |
| must have | any of | procedure | UMLS:CPT:31500 | Intubation, endotracheal, emergency procedure (at least 18 years old at event) |
| | | procedure | UMLS:ICD10PCS:0BH17EZ | Insertion of Endotracheal Airway into Trachea, Via Natural or Artificial Opening (at least 18 years old at event) |
| date constraint | | The terms in this group occurred between Jan 1, 2020 and Jun 30, 2025 | | |
| event relationship | | Any instance of exclude chronic adrenal insufficiency occurred within 1 year and 1 day before the first instance of Intubation | | |
| **Group 12B Exclude chronic adrenal insufficiency** | | | | |
| cannot have | | diagnosis | UMLS:ICD10CM:E27.1 | Primary adrenocortical insufficiency |
| | or | diagnosis | UMLS:ICD10CM:E27.2 | Addisonian crisis |
| | or | diagnosis | UMLS:ICD10CM:E27.3 | Drug-induced adrenocortical insufficiency |
| | or | diagnosis | UMLS:ICD10CM:E27.40 | Unspecified adrenocortical insufficiency |
| | or | diagnosis | UMLS:ICD10CM:E27.49 | Other adrenocortical insufficiency |
| **Group 13** | | | | |
| **Group 13A Intubation** | | | | |
| must have | any of | procedure | UMLS:CPT:31500 | Intubation, endotracheal, emergency procedure (at least 18 years old at event) |
| | | procedure | UMLS:ICD10PCS:0BH17EZ | Insertion of Endotracheal Airway into Trachea, Via Natural or Artificial Opening (at least 18 years old at event) |
| date constraint | | The terms in this group occurred between Jan 1, 2020 and Jun 30, 2025 | | |
| event relationship | | Any instance of exclude vasopressors occurred exactly 1 day before the first instance of Intubation | | |
| **Group 13B Exclude Vasopressors** | | | | |
| cannot have | | medication | NLM:RXNORM:7512 | norepinephrine |
| | or | medication | NLM:RXNORM:3992 | epinephrine |
| | or | medication | NLM:RXNORM:3628 | dopamine |
| | or | medication | NLM:RXNORM:11149 | vasopressin (USP) |
| | or | medication | NLM:RXNORM:8163 | phenylephrine |

**Abbreviations:** HCO, healthcare organization; NMB, neuromuscular blockade.

Note on stratified cohorts: The query criteria above define the primary matched cohort. For the hemodynamically stable cohort, Group 13 was modified to exclude vasopressor use on both day -1 and day 0 (rather than only on day -1). For the critically ill cohort, Group 13 was reversed to require vasopressor use on both day -1 and day 0. All other query criteria (Groups 1-12) were identical across all three cohorts.

**Supplementary Table S2. Query Criteria for Cohort 2 (query name: Ketamine)**

This query was run on the network US Collaborative Network with 69 HCO(s) queried and 69 HCO(s) responded. A total of 56 provider(s) responded with patients.

| | | | | | |
|---|---|---|---|---|---|
| **Group 1** | | | | | |
| **Intubation** | | | | | |
| must have | any of | procedure | UMLS:CPT:31500 | Intubation, endotracheal, emergency procedure (at least 18 years old at event) | |
| | | procedure | UMLS:ICD10PCS:0BH17EZ | Insertion of Endotracheal Airway into Trachea, Via Natural or Artificial Opening (at least 18 years old at event) | |
| date constraint | | The terms in this group occurred between Jan 1, 2020 and Jun 30, 2025 | | | |
| **Group 2** | | | | | |
| **Group 2A Intubation** | | | | | |
| must have | any of | procedure | UMLS:CPT:31500 | Intubation, endotracheal, emergency procedure (at least 18 years old at event) | |
| | | procedure | UMLS:ICD10PCS:0BH17EZ | Insertion of Endotracheal Airway into Trachea, Via Natural or Artificial Opening (at least 18 years old at event) | |
| date constraint | | The terms in this group occurred between Jan 1, 2020 and Jun 30, 2025 | | | |
| event relationship | | Any instance of confirmed COVID-19 infection occurred within 14 days on or before the first instance of Intubation | | | |
| **Group 2B Confirmed COVID-19 infection** | | | | | |
| must have | any of | diagnosis | UMLS:ICD10CM:U07.1 | COVID-19 | |
| | | laboratory | TNX:9088 | SARS coronavirus 2 and related RNA [Presence] | |
| | | laboratory | UMLS:LNC:94500-6 | SARS-CoV-2 (COVID-19) RNA [Presence] in Respiratory system specimen by NAA with probe detection | |
| | | laboratory | UMLS:LNC:94502-2 | SARS-related coronavirus RNA [Presence] in Respiratory system specimen by NAA with probe detection | |
| | | laboratory | UMLS:LNC:94565-9 | SARS-CoV-2 (COVID-19) RNA [Presence] in Nasopharynx by NAA with non-probe detection | |
| | | laboratory | UMLS:LNC:95209-3 | SARS-CoV+SARS-CoV-2 (COVID-19) Ag [Presence] in Respiratory system specimen by Rapid immunoassay | |
| | | laboratory | UMLS:LNC:96119-3 | SARS-CoV-2 (COVID-19) Ag [Presence] in Upper respiratory specimen by Immunoassay | |

| | | | | |
|---|---|---|---|---|
| | | laboratory | UMLS:LNC:94309-2 | SARS-CoV-2 (COVID-19) RNA [Presence] in Specimen by NAA with probe detection |
| | | laboratory | UMLS:LNC:94306-8 | SARS-CoV-2 (COVID-19) RNA panel - Specimen by NAA with probe detection |
| | | laboratory | UMLS:LNC:94558-4 | SARS-CoV-2 (COVID-19) Ag [Presence] in Respiratory system specimen by Rapid immunoassay |
| | | laboratory | UMLS:LNC:94759-8 | SARS-CoV-2 (COVID-19) RNA [Presence] in Nasopharynx by NAA with probe detection |
| | | laboratory | UMLS:LNC:94845-5 | SARS-CoV-2 (COVID-19) RNA [Presence] in Saliva (oral fluid) by NAA with probe detection |
| | | laboratory | UMLS:LNC:95406-5 | SARS-CoV-2 (COVID-19) RNA [Presence] in Nose by NAA with probe detection |
| | | laboratory | UMLS:LNC:97097-0 | SARS-CoV-2 (COVID-19) Ag [Presence] in Upper respiratory specimen by Rapid immunoassay |
| **Group 3** | | | | |
| **Group 3A Intubation** | | | | |
| must have | any of | procedure | UMLS:CPT:31500 | Intubation, endotracheal, emergency procedure (at least 18 years old at event) |
| | | procedure | UMLS:ICD10PCS:0BH17EZ | Insertion of Endotracheal Airway into Trachea, Via Natural or Artificial Opening (at least 18 years old at event) |
| date constraint | | The terms in this group occurred between Jan 1, 2020 and Jun 30, 2025 | | |
| event relationship | | Any instance of COVID-19 respiratory complications occurred within 14 days on or before the first instance of Intubation | | |
| **Group 3B COVID-19 respiratory complications** | | | | |
| must have | any of | diagnosis | UMLS:ICD10CM:J12.82 | Pneumonia due to coronavirus disease 2019 |
| | | diagnosis | UMLS:ICD10CM:J12.9 | Viral pneumonia, unspecified |
| | | diagnosis | UMLS:ICD10CM:J80 | Acute respiratory distress syndrome |
| | | diagnosis | UMLS:ICD10CM:J96.0 | Acute respiratory failure |
| **Group 4** | | | | |
| **Group 4A Intubation** | | | | |
| must have | any of | procedure | UMLS:CPT:31500 | Intubation, endotracheal, emergency procedure (at least 18 years old at event) |
| | | procedure | UMLS:ICD10PCS:0BH17EZ | Insertion of Endotracheal Airway into Trachea, Via Natural or Artificial Opening (at least 18 years old at event) |
| date constraint | | The terms in this group occurred between Jan 1, 2020 and Jun 30, 2025 | | |
| event relationship | | Any instance of NMB occurred on the same date as the first instance of Intubation | | |
| **Group 4B NMB** | | | | |
| must have | any of | medication | NLM:RXNORM:10154 | succinylcholine |
| | | medication | NLM:RXNORM:68139 | rocuronium |
| **Group 5** | | | | |

| | | | | |
|---|---|---|---|---|
| **Group 5A Intubation** | | | | |
| must have | any of | procedure | UMLS:CPT:31500 | Intubation, endotracheal, emergency procedure (at least 18 years old at event) |
| | | procedure | UMLS:ICD10PCS:0BH17EZ | Insertion of Endotracheal Airway into Trachea, Via Natural or Artificial Opening (at least 18 years old at event) |
| date constraint | | The terms in this group occurred between Jan 1, 2020 and Jun 30, 2025 | | |
| event relationship | | Any instance of ketamine occurred on the same date as the first instance of Intubation | | |
| **Group 5B Ketamine** | | | | |
| must have | | medication | NLM:RXNORM:6130 | ketamine |
| **Group 6** | | | | |
| **Group 6A Intubation** | | | | |
| must have | any of | procedure | UMLS:CPT:31500 | Intubation, endotracheal, emergency procedure (at least 18 years old at event) |
| | | procedure | UMLS:ICD10PCS:0BH17EZ | Insertion of Endotracheal Airway into Trachea, Via Natural or Artificial Opening (at least 18 years old at event) |
| date constraint | | The terms in this group occurred between Jan 1, 2020 and Jun 30, 2025 | | |
| event relationship | | Any instance of exclude etomidate occurred on the same date as the first instance of Intubation | | |
| **Group 6B Exclude etomidate** | | | | |
| cannot have | | medication | NLM:RXNORM:4177 | etomidate |
| **Group 7** | | | | |
| **Group 7A Intubation** | | | | |
| must have | any of | procedure | UMLS:CPT:31500 | Intubation, endotracheal, emergency procedure (at least 18 years old at event) |
| | | procedure | UMLS:ICD10PCS:0BH17EZ | Insertion of Endotracheal Airway into Trachea, Via Natural or Artificial Opening (at least 18 years old at event) |
| date constraint | | The terms in this group occurred between Jan 1, 2020 and Jun 30, 2025 | | |
| event relationship | | Any instance of exclude ketamine administration after occurred exactly 1 day after the first instance of Intubation | | |
| **Group 7B Exclude ketamine administration after the index day** | | | | |
| cannot have | | medication | NLM:RXNORM:6130 | ketamine |
| **Group 8** | | | | |
| **Group 8A Intubation** | | | | |
| must have | any of | procedure | UMLS:CPT:31500 | Intubation, endotracheal, emergency procedure (at least 18 years old at event) |
| | | procedure | UMLS:ICD10PCS:0BH17EZ | Insertion of Endotracheal Airway into Trachea, Via Natural or Artificial Opening (at least 18 years old at event) |
| date constraint | | The terms in this group occurred between Jan 1, 2020 and Jun 30, 2025 | | |
| event relationship | | Any instance of exclude ketamine administration before occurred exactly 1 day before the first instance of Intubation | | |

| | | | | | |
|---|---|---|---|---|---|
| | **Group 8B Exclude ketamine administration before the index day** | | | | |
| | cannot have | | medication | NLM:RXNORM:6130 | ketamine |
| **Group 9** | | | | | |
| | **Group 9A Intubation** | | | | |
| | must have | any of | procedure | UMLS:CPT:31500 | Intubation, endotracheal, emergency procedure (at least 18 years old at event) |
| | | | procedure | UMLS:ICD10PCS:0BH17EZ | Insertion of Endotracheal Airway into Trachea, Via Natural or Artificial Opening (at least 18 years old at event) |
| | date constraint | | The terms in this group occurred between Jan 1, 2020 and Jun 30, 2025 | | |
| | event relationship | | Any instance of no pregnancy occurred within 3 months on or before the first instance of Intubation | | |
| | **Group 9B No pregnancy** | | | | |
| | cannot have | | procedure | UMLS:ICD10PCS:10 | Pregnancy |
| | | or | diagnosis | UMLS:ICD10CM:O00-O9A | Pregnancy, childbirth and the puerperium |
| | | or | diagnosis | UMLS:ICD10CM:Z33.1 | Pregnant state, incidental |
| | | or | diagnosis | UMLS:ICD10CM:Z34 | Encounter for supervision of normal pregnancy |
| | | or | diagnosis | UMLS:ICD10CM:Z3A | Weeks of gestation |
| **Group 10** | | | | | |
| | **Group 10A Intubation** | | | | |
| | must have | any of | procedure | UMLS:CPT:31500 | Intubation, endotracheal, emergency procedure (at least 18 years old at event) |
| | | | procedure | UMLS:ICD10PCS:0BH17EZ | Insertion of Endotracheal Airway into Trachea, Via Natural or Artificial Opening (at least 18 years old at event) |
| | date constraint | | The terms in this group occurred between Jan 1, 2020 and Jun 30, 2025 | | |
| | event relationship | | Any instance of anesthesia occurred on the same date as the first instance of Intubation | | |
| | **Group 10B Anesthesia** | | | | |
| | cannot have | | procedure | UMLS:CPT:1002796 | Anesthesia |
| **Group 11** | | | | | |
| | **Group 11A Intubation** | | | | |
| | must have | any of | procedure | UMLS:CPT:31500 | Intubation, endotracheal, emergency procedure (at least 18 years old at event) |
| | | | procedure | UMLS:ICD10PCS:0BH17EZ | Insertion of Endotracheal Airway into Trachea, Via Natural or Artificial Opening (at least 18 years old at event) |
| | date constraint | | The terms in this group occurred between Jan 1, 2020 and Jun 30, 2025 | | |
| | event relationship | | Any instance of exclude previous tracheostomy occurred within 1 year and 1 day before the first instance of Intubation | | |
| | **Group 11B Exclude previous tracheostomy** | | | | |
| | cannot have | | diagnosis | UMLS:ICD10CM:Z93.0 | Tracheostomy status |

| | | | | |
|---|---|---|---|---|
| | or | procedure | UMLS:CPT:31600 | Tracheostomy, planned (separate procedure) |
| | or | procedure | UMLS:CPT:31603 | Tracheostomy, emergency procedure; transtracheal |
| | or | procedure | UMLS:CPT:31610 | Tracheostomy, fenestration procedure with skin flaps |
| **Group 12** | | | | |
| **Group 12A Intubation** | | | | |
| must have | any of | procedure | UMLS:CPT:31500 | Intubation, endotracheal, emergency procedure (at least 18 years old at event) |
| | | procedure | UMLS:ICD10PCS:0BH17EZ | Insertion of Endotracheal Airway into Trachea, Via Natural or Artificial Opening (at least 18 years old at event) |
| date constraint | | The terms in this group occurred between Jan 1, 2020 and Jun 30, 2025 | | |
| event relationship | | Any instance of exclude chronic adrenal insufficiency occurred within 1 year and 1 day before the first instance of Intubation | | |
| **Group 12B Exclude chronic adrenal insufficiency** | | | | |
| cannot have | | diagnosis | UMLS:ICD10CM:E27.1 | Primary adrenocortical insufficiency |
| | or | diagnosis | UMLS:ICD10CM:E27.2 | Addisonian crisis |
| | or | diagnosis | UMLS:ICD10CM:E27.3 | Drug-induced adrenocortical insufficiency |
| | or | diagnosis | UMLS:ICD10CM:E27.40 | Unspecified adrenocortical insufficiency |
| | or | diagnosis | UMLS:ICD10CM:E27.49 | Other adrenocortical insufficiency |
| **Group 13** | | | | |
| **Group 13A Intubation** | | | | |
| must have | any of | procedure | UMLS:CPT:31500 | Intubation, endotracheal, emergency procedure (at least 18 years old at event) |
| | | procedure | UMLS:ICD10PCS:0BH17EZ | Insertion of Endotracheal Airway into Trachea, Via Natural or Artificial Opening (at least 18 years old at event) |
| date constraint | | The terms in this group occurred between Jan 1, 2020 and Jun 30, 2025 | | |
| event relationship | | Any instance of exclude vasopressors occurred exactly 1 day before the first instance of Intubation | | |
| **Group 13B Exclude Vasopressors** | | | | |
| cannot have | | medication | NLM:RXNORM:7512 | norepinephrine |
| | or | medication | NLM:RXNORM:3992 | epinephrine |
| | or | medication | NLM:RXNORM:3628 | dopamine |
| | or | medication | NLM:RXNORM:11149 | vasopressin (USP) |
| | or | medication | NLM:RXNORM:8163 | phenylephrine |

**Abbreviations:** HCO, healthcare organization; NMB, neuromuscular blockade.

Note on stratified cohorts: The query criteria above define the primary matched cohort. For the hemodynamically stable cohort, Group 13 was modified to exclude vasopressor use on both day -1 and day 0 (rather than only on day -1). For the critically ill cohort, Group 13 was reversed to require vasopressor use on both day -1 and day 0. All other query criteria (Groups 1-12) were identical across all three cohorts.

## Supplementary Table S3. Propensity Score Matching by using following parameters

| Etomidate and ketamine characteristics after propensity score matching | |
|---|---|
| **Demographics** | |
| **Code** | **Name** |
| AI | Age at Index |
| M | Male |
| F | Female |
| *Race* | |
| 2106-3 | White |
| 2054-5 | African American |
| 2028-9 | Asian |
| 2131-1 | Other Race |
| UNK | Unknown Race |
| *Ethnicity* | |
| 2186-5 | Not Hispanic or Latino |
| 2135-2 | Hispanic or Latino |
| UN | Unknown Ethnicity |
| **Comorbidity** | |
| **ICD-10-CM** | **Name** |
| E08-E13 | Diabetes mellitus |
| E66 | Overweight and obesity |
| I10-I1A | Hypertensive diseases |
| I20-I25 | Ischemic heart diseases |
| I48 | Atrial fibrillation and flutter |
| I50 | Heart failure |
| I60-I69 | Cerebrovascular diseases |
| J40-J4A | Chronic lower respiratory diseases |
| K72 | Hepatic failure |
| K74 | Liver cirrhosis |
| K70 | Alcoholic liver disease |
| N18.3 | Chronic kidney disease, stage 3 |
| N18.4 | Chronic kidney disease, stage 4 |
| N18.5 | Chronic kidney disease, stage 5 |
| N18.6 | End stage renal disease |
| S06 | Intracranial injury |
| *Malignancies** | |
| C69-C72 | Central nerve system |
| C00-C14 | Head/neck |
| C30-C39 | Respiratory/thoracic |
| C15-C26 | Gastrointestinal |
| C64-C68 | Urinary tract |
| C60-C63 | Urologic, male |
| C51-C58 | Gynecologic |
| C50 | Breast |
| C81-C96 | Hematologic |
| C40-C41 | Bone/cartilage |
| C43-C44 | Skin/melanoma |
| C45-C49 | Mesothelioma/soft tissue |
| C7A | Neuroendocrine |
| C73-C75 | Thyroid/other endocrine |
| C76-C80 | Other/unspecified |

| **Medication** | | |
|---|---|---|
| **RxNorm** | **Name** | |
| 10154 | Succinylcholine | |
| 68139 | Rocuronium | |
| 213 | SARS-CoV-2 vaccine | |
| 3264 | Dexamethasone | |
| 8638 | Prednisolone | |
| 2284718 | Remdesivir | |
| 2047232 | Baricitinib | |
| 612865 | Tocilizumab | |
| 2587892 | Nirmatrelvir | |
| 85762 | Ritonavir | |
| 2587901 | Molnupiravir | |
| **Clinical parameters** | | |
| **TNX Code** | **Name** | **Unit** |
| 9083 | BMI | $kg/m^2$ |
| 9073 | Respiratory rate | breaths/min |
| 9074 | Heart rate | beats/min |
| 9075 | Oxygen saturation | % |
| 9085 | Systolic blood pressure | mmHg |
| 9086 | Diastolic blood pressure | mmHg |
| 9076 | Body temperature | °F |
| **Laboratory tests** | | |
| **TNX Code** | **Name** | **Unit** |
| 9014 | Hemoglobin | g/dL |
| 9015 | Leukocytes | $\times 10^3/\mu L$ |
| 9020 | Platelets | $\times 10^3/\mu L$ |
| 9018 | Neutrophils | $\times 10^3/\mu L$ |
| 9016 | Lymphocytes/100 leukocytes | % |
| 9030 | Urea nitrogen | mg/dL |
| 9024 | Creatinine | mg/dL |
| 9029 | Sodium | mmol/L |
| 9028 | Potassium | mmol/L |
| 9021 | Bicarbonate | mmol/L |
| 9025 | Glucose | mg/dL |
| 9044 | Alanine aminotransferase | U/L |
| 9047 | Aspartate aminotransferase | U/L |
| 9050 | Bilirubin, total | mg/dL |
| 9048 | Bilirubin, direct | mg/dL |
| 9033 | Prothrombin time | s |
| 9031 | Activated partial thromboplastin time | s |
| 48065-7 | Fibrin D-dimer FEU | mg/L |
| 9063 | C-reactive protein | mg/dL |
| LG15749-1 | Procalcitonin | ng/mL |
| 9066 | Erythrocyte sedimentation rate | mm/h |
| 9005 | Troponin I | ng/mL |
| 9003 | Natriuretic peptide B | pg/mL |
| 9068 | Lactate | mmol/L |

***Malignancies:** Central nerve system = malignant neoplasms of eye, brain and other parts of central nervous system; Head/neck = malignant neoplasms of lip, oral cavity and pharynx; Respiratory/thoracic = malignant neoplasms of respiratory and intrathoracic organs; Gastrointestinal = malignant neoplasms of digestive organs; Urinary tract = malignant neoplasms of urinary tract; Urologic, male = malignant neoplasms of male genital organs; Gynecologic = malignant neoplasms of female genital organs; Breast = malignant neoplasms of breast; Hematologic

= malignant neoplasms of lymphoid, hematopoietic and related tissue; Bone/cartilage = malignant neoplasms of bone and articular cartilage; Skin/melanoma = melanoma and other malignant neoplasms of skin; Mesothelioma/soft tissue = malignant neoplasms of mesothelial and soft tissue; Neuroendocrine = malignant neuroendocrine tumors; Thyroid/other endocrine = malignant neoplasms of thyroid and other endocrine glands; Other/unspecified = malignant neoplasms of ill-defined, other secondary and unspecified sites.

**Abbreviations:** ICD-10-CM, International Classification of Diseases, Tenth Revision, Clinical Modification; BMI, body mass index; FEU, fibrinogen equivalent units.

**Supplementary Table S4. Outcome Definitions**

Table below outlines the definitions for each outcome and the analysis specifications. For outcome definitions consisting of more than one term, at least one term must match.

| | | | |
|---|---|---|---|
| **Mortality** | | | |
| **Outcome definition** | | | |
| | Demographics | Deceased | Deceased |
| | Risk analysis | | including patients with outcome prior to the time window |
| **Vasopressors (excl. epinephrine)** | | | |
| **Outcome definition** | | | |
| | Medication | NLM:RXNORM:7512 | norepinephrine |
| | Medication | NLM:RXNORM:3628 | dopamine |
| | Medication | NLM:RXNORM:11149 | vasopressin (USP) |
| | Medication | NLM:RXNORM:8163 | phenylephrine |
| **Adrenal insufficiency** | | | |
| **Outcome definition** | | | |
| | Diagnosis | UMLS:ICD10CM:E27.1 | Primary adrenocortical insufficiency |
| | Diagnosis | UMLS:ICD10CM:E27.2 | Addisonian crisis |
| | Diagnosis | UMLS:ICD10CM:E27.3 | Drug-induced adrenocortical insufficiency |
| | Diagnosis | UMLS:ICD10CM:E27.4 | Other and unspecified adrenocortical insufficiency |
| | Laboratory | TNX:9035 | Cortisol [Mass/volume] in Serum or Plasma (at most 9.90 ug/dL (most recent occurrence)) |
| **Cystitis** | | | |
| **Outcome definition** | | | |
| | Diagnosis | UMLS:ICD10CM:N30 | Cystitis |
| **Cellulitis** | | | |
| **Outcome definition** | | | |
| | Diagnosis | UMLS:ICD10CM:L03 | Cellulitis and acute lymphangitis |

**Supplementary Figure S1. Vasopressor initiation in the primary matched cohort.**

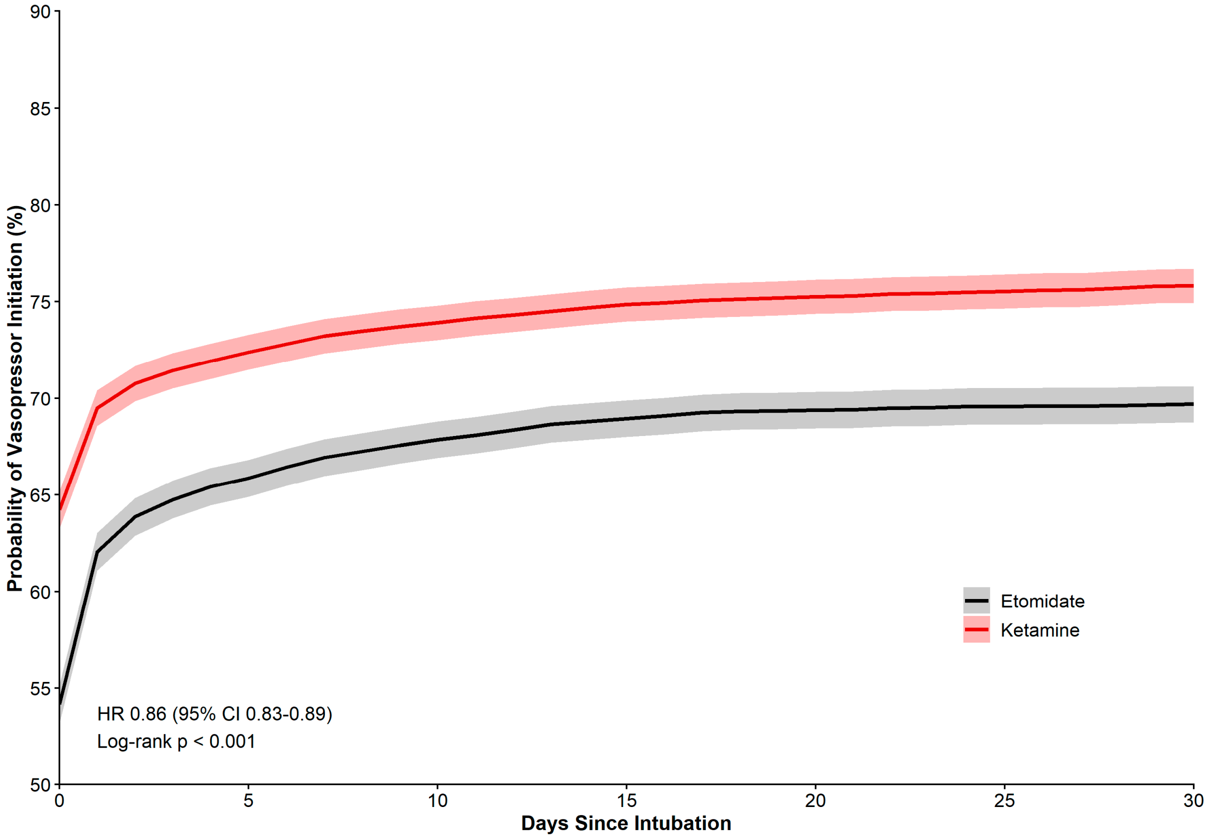


**Supplementary Figure S1.** Kaplan-Meier curves showing cumulative incidence of vasopressor initiation (excluding epinephrine) over 30 days in the primary matched cohort (n=9,462 per group). Etomidate (black line, gray shaded 95% CI band) and ketamine (red line, pink shaded 95% CI band) curves were generated using the vasopressor-Epi outcome (norepinephrine, dopamine, vasopressin, or phenylephrine; epinephrine excluded to focus on sustained hemodynamic support). By the end of day 0, vasopressor initiation had reached 54.17% in the etomidate group and 64.23% in the ketamine group—a 10-percentage-point difference established within day 0 itself. By day 30, vasopressor initiation had risen to 69.69% in the etomidate group and 75.81% in the ketamine group, with the between-group difference narrowing to approximately 6 percentage points. Hazard ratio 0.86 (95% CI 0.83–0.89, log-rank p<0.001). Y-axis range 50–90%; vasopressor initiation began at 0% on day 0 (before intubation).
